# Environmental Temperature and Thermal Indices: What Is the Most Effective Predictor of Heat-Related Mortality in Different Geographical Contexts?

**DOI:** 10.1155/2014/961750

**Published:** 2014-01-08

**Authors:** Marco Morabito, Alfonso Crisci, Alessandro Messeri, Valerio Capecchi, Pietro Amedeo Modesti, Gian Franco Gensini, Simone Orlandini

**Affiliations:** ^1^Department of Agrifood Production and Environmental Sciences, University of Florence, Piazzale delle Cascine 18, 50144 Florence, Italy; ^2^Interdepartmental Centre of Bioclimatology, University of Florence, Piazzale delle Cascine 18, 50144 Florence, Italy; ^3^Center for Civil Protection and Risk Studies, University of Florence (CESPRO), Viale Morgagni 48, 50134 Florence, Italy; ^4^Institute of Biometeorology, National Research Council, Via Giovanni Caproni 8, 50145 Florence, Italy; ^5^Clinica Medica e Cardiologia, University of Florence, Viale Morgagni 85, 50134 Florence, Italy

## Abstract

The aim of this study is to identify the most effective thermal predictor of heat-related very-elderly mortality in two cities located in different geographical contexts of central Italy. We tested the hypothesis that use of the state-of-the-art rational thermal indices, the Universal Thermal Climate Index (UTCI), might provide an improvement in predicting heat-related mortality with respect to other predictors. Data regarding very elderly people (≥75 years) who died in inland and coastal cities from 2006 to 2008 (May–October) and meteorological and air pollution were obtained from the regional mortality and environmental archives. Rational (UTCI) and direct thermal indices represented by a set of bivariate/multivariate apparent temperature indices were assessed. Correlation analyses and generalized additive models were applied. The Akaike weights were used for the best model selection. Direct multivariate indices showed the highest correlations with UTCI and were also selected as the best thermal predictors of heat-related mortality for both inland and coastal cities. Conversely, the UTCI was never identified as the best thermal predictor. The use of direct multivariate indices, which also account for the extra effect of wind speed and/or solar radiation, revealed the best fitting with all-cause, very-elderly mortality attributable to heat stress.

## 1. Introduction

The relationship between high environmental temperature and human mortality has been widely investigated and at the present time a large amount of scientific studies and detailed reviews are available for people living in different geographical areas [1–5]. However, the correct interpretation and comparison of results from environmental epidemiological studies are not immediate because different thermal/temperature based health-impact indicators have been adopted. For example, several authors used air temperature variables (such as daily average, maximum, and minimum air temperature) as environmental predictors of human mortality [3, 6, 7], while others used alternative temperature metrics which condense all the extra meteorological effects (i.e., air humidity, wind speed, and solar radiation) into a single number derived by means of more or less complex thermal indices [8–11]. Thermal indices are useful tools for summarizing the interaction of thermal environmental stressors on humans. Thermal indices can be categorized by direct (based on direct measurements of environmental variables), empirical (based on objective and subjective stress), or rational (based on calculations involving the human heat balance) indices [12]. Most studies examined to evaluate the impact of heat stress on mortality mainly used direct indices, especially because they are quick and easy to use and usually take into account the combined effect of only two main meteorological variables for thermal comfort evaluations (air temperature and humidity), normally available from regular measurements of typical weather stations. Further direct indices also allow for including the combined effect of other environmental variables, such as wind speed and occasionally, depending on data availability, solar radiation [13], both of which are important for the outdoor thermal comfort assessment. However, at the present time, the application of these indices in the field of heat-related mortality is very rare [14]. While only a small number of epidemiological studies have ever attempted to use more complex and complete rational indices [15], the newly developed Universal Thermal Climate Index (UTCI) [16] that represents the state-of-the-art in outdoor thermal comfort assessments has never been employed.

In a recent study [12], several authors reported a detailed comparison of UTCI with a selected set of thermal indices and stated that direct indices (i.e., apparent temperature) are less correlated with UTCI than other indices derived from the human heat budget model. The authors claimed that one of the possible causes of unconformity is the lack of the radiation factor in the algorithm equations.

Currently, it is very difficult to compare the results of heat-related mortality from studies that used different predictors such as air temperature variables or thermal indices, and several significant doubts regarding the application of one or other environmental indicator exist among researchers involved in environmental epidemiological studies. In particular, what is the correlation pattern among different temperature and thermal index indicators, especially when the results in geographical areas are compared with very different weather conditions (i.e., strong winds, high humidity, etc.)? Consequently, what is the difference in the predicted heat-related mortality if different temperature variables or thermal index indicators are considered? Are there significant differences when different climatic conditions and geographical locations are taken into account? In short, what is the most effective thermal indicator of heat-related mortality?

In previous studies [14, 17–19] the authors tried to address this issue but only simple thermal indices (generally thermohygrometric indices) were considered and the UTCI was never taken into account.

For this reason, the main aim of this study is to identify the most effective thermal/air temperature indicators for predicting heat-related mortality of the very elderly in two cities with different geographical characteristics based mainly on their distance from the Tyrrhenian Sea (coastal and inland plain cities). We put the hypothesis to the test that the use of the state of the art to assess outdoor thermal comfort/discomfort (UTCI) might provide an effective improvement in predicting heat-related mortality with respect to direct thermal indices or simple air temperature variables currently used in the literature. Furthermore, the potentially different impact on mortality due to environmental heat conditions is also investigated by using two types of meteorological data sources coming from urban and suburban weather stations. This information could prove to be very useful in developing preventive measures and for implementing local public health emergency plans related to heat-stress conditions.

## 2. Material and Methods

### 2.1. Mortality Data and Study Area

The health outcome data consisted of residents of the two major inland (Florence) and coastal (Livorno) cities in the Tuscany region (Central Italy) who died of nonviolent causes during the hottest period of the year (May 1 to October 31) from 2006 to 2008. Non-accidental mortality data (ICD9 < 800) were provided by the Mortality Registry of the Tuscany region. Very elderly residents (≥75 years old) who died of nonviolent causes in the two cities were selected for the analyses (*n* = 3,852  in Florence and *n* = 1,942 in Livorno).

The cities considered in this study are located in different geographical contexts in terms of morphological and climatic conditions. (a) Florence is an inland plain city located 80 km from the Tyrrhenian Sea at an average altitude of 50 m a.s.l. (lat. 43°46′17′′N; long. 11°15′15′′E). The average urban population density for the 3-year period studied was 3,570 inhabitants per km^2^ (the highest population density in the Tuscany region). The percentage of the very elderly (age ≥ 75 years) population was 13.6%. (b) Livorno is a coastal plain city at about 10 m a.s.l. with its port on the Tyrrhenian Sea (lat. 43°33′0′′N; long. 10°19′0′′E). The average urban population density was 1,540 inhabitants per km^2^ (the highest population density in coastal Tuscan cities). The percentage of the very elderly population was 12.1%.

In regard to the climatic features of the areas studied, July and August are the warmest months in both cities investigated, while the coldest months are December and January. The inland plain city is characterized by higher/lower temperatures than the coastal plain city. Furthermore, there is a wide daily temperature range in the inland city and during the warmest months. Conversely, the coastal plain city is generally characterized by a milder climate due to its close vicinity to the Tyrrhenian Sea, and it also has the shortest daily temperature range with rare extreme temperatures.

### 2.2. Meteorological and Environmental Pollution Data

Hourly meteorological data regarding air temperature (*T*_air_, °C), relative humidity (RH, %), 10 m high horizontal wind speed (*V*_10_, m s^−1^), and global radiation (GR, W m^−2^) were provided by four meteorological stations managed by the Regional Weather Service of Tuscany. Meteorological data covered the warmest period of the year (May–October) from 2006 to 2008.

Two of these meteorological stations were located in urban districts, and in particular, in two green areas of the city centers of Florence and Livorno. The other two stations were located in residential districts in the flat north-west area of Florence and on the coast (about 100 m from the sea) of Livorno.

For the same period, air pollution data, including daily average values of ambient particulate concentrations with aerodynamic diameter ≤10 *μ*m (PM_10_, *μ*g m^−3^), nitrogen dioxide (NO_2_, *μ*g m^−3^), sulfur dioxide (SO_2_, *µ*g m^−3^), carbon monoxide (CO, mg m^−3^), and ozone (O_3_, *µ*g m^−3^), were obtained from the Environmental Protection Agency of Tuscany. Daily pollutant concentrations were averaged from available monitoring stations located in each urban and suburban area of Florence and Livorno. The number of monitoring sites generally varied from two to five pollution stations depending on the pollutant monitored.

During the study period, the methods and instruments of the monitoring sites, classified as “urban background” (based on European and Italian air quality legislation), were homogeneous and compliant with the quality assurance criteria. Furthermore, based on previous studies in the same geographical areas [20, 21], the selected environmental monitoring sites showed homogeneous air-quality levels and offered good representation of the background exposure of the general population in urban areas.

### 2.3. Biometeorological Indices Assessment

Three direct biometeorological indices (apparent temperature indices derived from Steadman's studies) and one rational index (Universal Thermal Climate Index) were assessed.

The apparent temperature (AT) is represented by a set of simple computational formulas which describe the combined effect of temperature and humidity, also taking into account the extra effects of wind speed and solar radiation, by measuring the thermal comfort of a typical human walking at 1.4 m s^−1^ and generating 177 W m^−2^ of total body surface [13, 22–24]. AT is always expressed in °C and for this reason it is easily interpreted by general users.

The three versions of apparent temperature (AT) indices used in this study are the following.

(i) The indoor AT (AT_ind_) only takes the combined effect of air temperature and humidity into consideration; this index is assessed by the following formula:
  (1) $$\begin{array}{c} {\text{AT}}_{\text{ind}}=0.89T_{\text{air}}+0.382e-2.56. \end{array}$$
(ii) The shade AT (AT_sha_) also takes the assessed wind effect into account; this index is assessed by the following formula:
  (2) $$\begin{array}{c} {\text{AT}}_{\text{sha}}=T_{\text{air}}+0.33e-0.70V_{10}-4.00. \end{array}$$
(iii) The outdoor AT (AT_sun_) expresses the sensation of a walking, clothed person fully exposed to all meteorological effects considered: air temperature and humidity, wind speed, and solar radiation; this index is assessed by the following formula:
  (3) $$\begin{array}{c} {\text{AT}}_{\text{sun}}=T_{\text{air}}+0.348e-0.70V_{10}+0.70\frac{Q_{g}}{\left(V_{10}+10\right)}-4.25, \end{array}$$
  where “*e*” is the water vapor pressure (hPa) and “*Q*_*g*_” is the heat-flow rate per unit area of body surface due to net extra radiation (*Q*_*g*_ is related to the mean radiant temperature). A detailed description of the assessment of *Q*_*g*_ is reported in Steadman (1994). In this study the variable “*e*” was calculated from the air temperature and the relative humidity using the following equation:
  (4) $$\begin{array}{c} e=\frac{\text{RH}}{100\;}6.105\exp^{\left(17.27(T_{\text{air}}/(237.7+T_{\text{air}}))\right)}. \end{array}$$

The Universal Thermal Climate Index (UTCI) is a comprehensive model fitted to assess human thermal comfort in outdoor environments and represents the state-of-the-art of outdoor thermal comfort indices [16]. UTCI is an equivalent temperature (°C) based on the most recent scientific progress in human thermo-physiology, biophysics, and the heat exchange theory [25]. The UTCI represents the efforts of a group of over 45 scientists from 23 countries collaborating together within the COST action 730 [16]. The advanced multinode dynamic UTCI-Fiala mathematical model of human temperature regulation forms the basis of the UTCI. Furthermore, the UTCI also includes a sophisticated clothing model that defines in detail the effective clothing insulation and vapor resistance values for each of the thermophysiological model's body segments over a wide range of climatic conditions. A detailed description of the UTCI is reported in Jendritzky et al. [16]. In this study, the UTCI was assessed by using the UTCI software code “version a 0.002”, freely available online (http://www.utci.org/). Currently, UTCI software uses fixed value for metabolic rate (activity level) and, depending on air temperature, also clothing insulation. The input parameters for the assessment of the UTCI that refers to a person walking at 4 km h^−1^ and generating 135 W m^−2^, are *T*_air_ (°C), *e* (hPa), *V*_10_ (m s^−1^), and the mean radiant temperature (*T*_mrt_, °C). The estimation of the *T*_mrt_ was carried out separately by using the RayMan software version 2.0 [26]. One of the aims of the RayMan model is to calculate short- and long-wave radiation flux densities absorbed by people that can be transferred into a synthetic parameter, that is *T*_mrt_, defined as the uniform temperature of a hypothetical spherical surface surrounding a human (emissivity *ε* = 1) which would result in the same net radiation energy exchange with the subject as the actual, complex radiative environment [26].

### 2.4. Statistical Analyses

A preliminary descriptive analysis was carried out of the characteristics of the daily mortality, air pollution, and main meteorological variables recorded in the two cities by urban and suburban weather stations during the warmest period of the year (May–October). In the following, a detailed description of daily summaries (daily average, maximum, and minimum) of thermal indices and air temperature indicators (defined as thermal indicators) was provided for both urban and suburban areas of the inland and coastal cities.

The analyses were organized in two main sections: (1) correlation analyses between the UTCI (rational index) versus direct indices and air temperature variables, assessed and measured by using urban and suburban meteorological stations; (2) investigation of relationships between daily mortality and the set of independent predictors represented by biometeorological (rational and direct indices) and meteorological (air temperature) indicators.

The first section of the study included linear regression analyses of daily average, maximum, and minimum UTCI in order to select direct daily biometeorological indices and air temperature variables. Statistical characteristics of the relationships were shown as slope coefficients of regressions and their relative *R*-squared (%).

The second section was investigated through a time-series approach by using generalized additive models (GAMs) [27]. The GAM approach is useful for detecting the temporal modification of heat-related mortality based on different thermal indicators. GAMs are very flexible tools that allow for applying a wide variety of link functions on the dependent variable for taking any non-linear (smooth) effects of predictor variables into account. A Poisson link assumption was used in this study.

The GAM procedure was performed using *R* software version 2.15.3 [28] and specifically the “mgcv” package [29]. GAM procedures were systematically used throughout the entire warmest period of the year to estimate the smoothed shape of exposure-response curves between total mortality and short-term changes of the set of daily average, maximum, and minimum independent biometeorological indices (UTCI, AT_sun_, AT_sha_, and AT_ind_) and air temperature (*T*_air_) variables, measured by using urban and suburban meteorological data. On the whole, 30 thermal indicators were identified for each city. The short-term change was calculated by averaging the daily biometeorological value or air temperature on a specific day together with the one calculated on the previous day (lag_0-1_).

GAM models were controlled for typical air pollution concentrations and calendar factor confounders such as daylight hours (that represent a proxy parameter of the season), year (to check for annual variation in mortality), day of the week, public holidays, and summer population decrement.

The final model specification is based on the following equation:

(5) $$\begin{array}{c} \ln\left(E\left(Y\right)\right)=\beta_{0}+\beta_{1}\cdot X_{t}+\Sigma S_{i}\left(X_{i}\right), \end{array}$$

where *E*(*Y*) is the estimated daily death count; *β*_0_ is the intercept of the regression; *β*_1_ is the coefficient (slope) for the thermal indicator (*X*_*t*_); *S*_*i*_(*X*_*i*_) denotes the smooth functions for the covariates (continuous: daylight hours and air pollutants; categorical: year, day of the week, public holidays, and summer population decrement).

Finally, in order to focus on the relationship between heat and mortality, the expected % change of death due to a 1°C increase in air temperature (or thermal index temperature) was assessed by selecting the upper 25% (75th percentile) of data based on each thermal indicator. The selection of the 75th percentile was based on an empirical assessment in model fittings, where overall daily death counts generally showed a monotonic increase as thermal indicators increased.

### 2.5. Model Selection

Akaike Information Criterion (AIC) [30] was applied to the entire warmest period for the model-fitting criteria. AIC represents one of the most reliable methods for comparing different models, taking both descriptive accuracy and parsimony into account [31]. AIC has been widely applied in many statistical fields or research including time series model selection [32].

The AIC is defined as [33]

(6) $$\begin{array}{c} {\text{AIC}}_{i}=-2\log L_{i}+2V_{i}, \end{array}$$

where *L*_*i*_ represents the maximum likelihood for a candidate model *i* and is determined by adjusting the *V*_*i*_ free parameters in such a way as to maximize the probability of the candidate model generating the observed data [31].

Given a set of candidate models for the data, it is well known that the preferred model is the one with the minimum AIC value, which is the model with the lowest expected information loss.

However, because from a statistical point of view it is difficult to understand the importance of the AIC difference, Δ_*i*_(AIC), between the best model (that is the model with the lowest AIC value), for example, and the next-best model (the second lowest AIC value), the AIC values obtained for each candidate model in this study have been transformed to the so-called Akaike weights, *w*_*i*_ (AIC) [33]. For this reason, all the AIC differences with respect to the AIC of the best candidate model were calculated as follows:

(7) $$\begin{array}{c} \Delta_{i}\left(\text{AIC}\right)={\text{AIC}}_{i}-\min\left(\text{AIC}\right). \end{array}$$

In the following, the Akaike weights were assessed by dividing the relative likelihood of a model *i* by the sum of the likelihoods of all models *k* as per the following equation:

(8) $$\begin{array}{c} w_{i}\left(\text{AIC}\right)=\frac{\exp\left\{-\left(1/2\right)\Delta_{i}\left(\text{AIC}\right)\right\}}{\sum_{k=1}^{K}\exp\left\{-\left(1/2\right)\Delta_{k}\left(\text{AIC}\right)\right\}}. \end{array}$$

In this way, the *w*_*i*_ (AIC) can be interpreted as the probability that a hypothetical model is the best predictive model among the set of candidate models. The best model is the one that minimizes the Kullback-Leibler discrepancy, which is a measure of the distance between the probability density generated by the model and reality [31].

Akaike weights quantify conclusions based on AIC analyses and provide a straightforward interpretation of the AIC model comparison analysis.

## 3. Results

### 3.1. Descriptive Statistics of Mortality, Air Pollution, and Meteorological/Biometeorological Data

The characteristics of daily all-cause mortality of people aged ≥75 years, mean air pollution concentrations, and meteorological variables recorded during the warmest period of the year (from May to October) are illustrated in Table 1.

Nonaccidental mortality data of the very elderly in the inland city was about twice as high as in the coastal city. Average NO_2_, PM_10_, and CO concentrations were significantly higher in the inland plain city than in the coastal one. On the other hand, the average O_3_ and especially SO_2_ showed the highest values in Livorno (Table 1).

Both the inland and coastal cities showed higher daily mean *T*_air_ in the suburbs than in the urban areas. Moreover, the *T*_air_ in the urban area of the inland plain city was slightly higher than the *T*_air_ in the coastal city. Conversely, the opposite situation was observed when the suburbs were considered. The *T*_air_ range over the warmest period of the year (differences between 90th and 10th percentiles of *T*_air_ data) was not as wide in urban areas and coastal city as in the suburbs and inland city. As expected, mean RH and *V*_10_ were always higher in the coastal city than in the inland one, with the highest values in the suburbs. The mean *T*_mrt_ was lower in the urban than suburban areas, with the highest mean values on the coast. Moreover, both inland and coastal cities showed wider *T*_mrt_ ranges than *T*_air_ ranges (Table 1).

Thermal indices and air temperature indicators in the coastal city generally showed higher mean values than in the inland city, with several exceptions, especially when daily maximum values in both urban and suburban areas (Table 2) were taken into account. More specifically, when the maximum *T*_air_ was considered, the lowest values were observed in the coastal city.

The daily mean average, minimum, and maximum thermal indicators measured in urban areas generally showed lower values than in the suburbs, with the only exception for the daily maximum UTCI in both cities and the maximum AT_sun_ in the coastal city, which recorded the opposite situation. Furthermore, thermal indicators in the suburbs of both Florence and Livorno always showed wider thermal ranges (differences between 90th and 10th percentiles of data) than those observed in urban areas (Table 2).

The AT_sun_ always disclosed the highest daily average, minimum, and maximum values among all indicators. On the other hand, UTCI and *T*_air_ often evidenced the lowest daily mean average and minimum values. The AT_sha_, which also takes into consideration the cooling effect of wind, always showed the lowest values among the direct indices. When daily maximum values were considered, both AT_sun_ and UTCI revealed the highest values when compared with the other indicators, thanks to the solar radiation contribution (Table 2).

### 3.2. Rational Index (UTCI) versus Direct Indices and Air Temperature Variables

The comparison between daily average, minimum, and maximum UTCI and direct bivariate/multivariate indices or air temperatures (Table 3) revealed that both AT_sun_ and AT_sha_ showed the highest correlation coefficients in both urban and suburban areas of the inland and coastal cities. On the other hand, both AT_ind_ and *T*_air_ showed the lowest *R* ^2^ coefficients.

Generally, AT_sun_ and AT_sha_ revealed that were the highest mean correlations when daily minimum values were considered, slightly higher than the correlations when daily average values were used. An opposite situation was observed when AT_ind_ and *T*_air_ were considered, whereas daily maximum values always showed the lowest correlation coefficients.

When daily average indicator values were considered, there was a progressive decrease of the mean *R* ^2^ coefficients (averaged from all stations), with the highest values observed for AT_sun_ (96.50%) followed by AT_sha_ (96.46%), AT_ind_ (93.34%), and *T*_air_ (93.19%) (Table 3). When daily minimum values were considered, the highest mean *R* ^2^ coefficient was observed for AT_sha_ (96.67%), slightly higher than the AT_sun_ (96.56%) value. When daily maximums were considered, *T*_air_ often showed a better fit than AT_ind_ and AT_sha_. However, *T*_air_ always showed the worst mean slope coefficients of regression lines, with the lowest values (from 0.50 to 0.56) observed in the suburb of the coastal city (Table 3). This indicates that UTCI and *T*_air_ change at different rates within various ranges of ambient conditions, especially in suburban areas.

Conversely, the best mean slope coefficients were always observed when AT_sun_ was considered: mean slope 1.00, 1.03, and 0.92 for average, minimum, and maximum values, respectively.

### 3.3. Relationships between Mortality and the Set of Biometeorological Indices and Air Temperature Indicators

**Figure 1:**
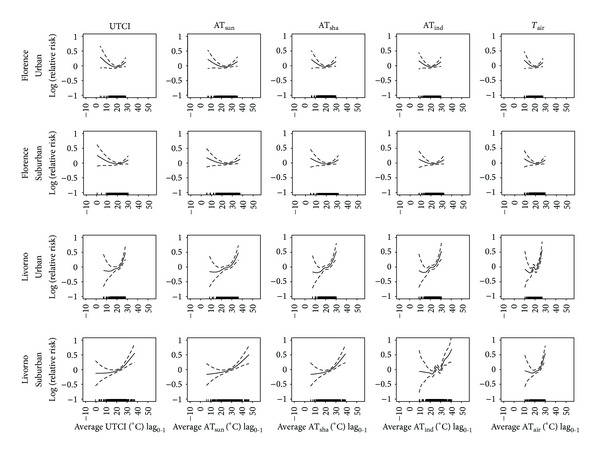
Relationships between daily average thermal predictors (lag_0-1_) (°C) and mortality of the very elderly (subjects ≥75 years of age) in an inland city (Florence) and coastal-plain city (Livorno). Relationships estimated by using urban and suburban meteorological data for the period 2006–2008 (May–October). UTCI: Universal Thermal Climate Index; AT_sun_: apparent temperature assessed outdoors also taking the solar radiation contribution into account; AT_sha_: apparent temperature assessed outdoors in the shade; AT_ind_: apparent temperature assessed in indoor conditions; *T*_air_: environmental temperature. Analyses were controlled for air pollution concentrations, daylight hours, year, day of the week, public holidays, and summer population decrement.

**Figure 2:**
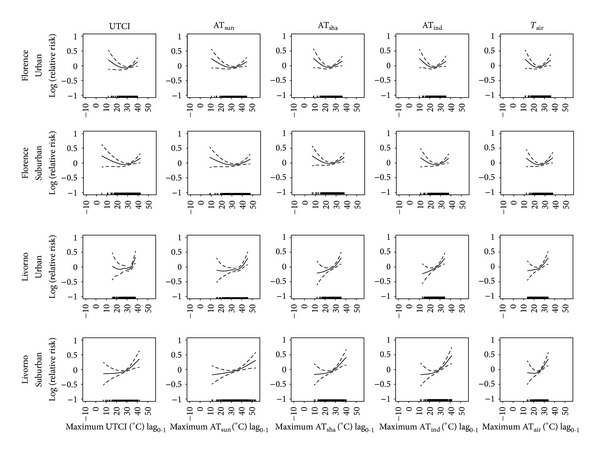
Relationships between daily maximum thermal predictors (lag_0-1_) (°C) and mortality of the very elderly (subjects ≥75 years of age) in an inland city (Florence) and coastal-plain city (Livorno). Relationships estimated by using urban and suburban meteorological data for the period 2006–2008 (May–October). UTCI: Universal Thermal Climate Index; AT_sun_: apparent temperature assessed outdoors also taking the solar radiation contribution into account; AT_sha_: apparent temperature assessed outdoors in the shade; AT_ind_: apparent temperature assessed in indoor conditions; *T*_air_: environmental temperature. Analyses were controlled for air pollution concentrations, daylight hours, year, day of the week, public holidays, and summer population decrement.

**Figure 3:**
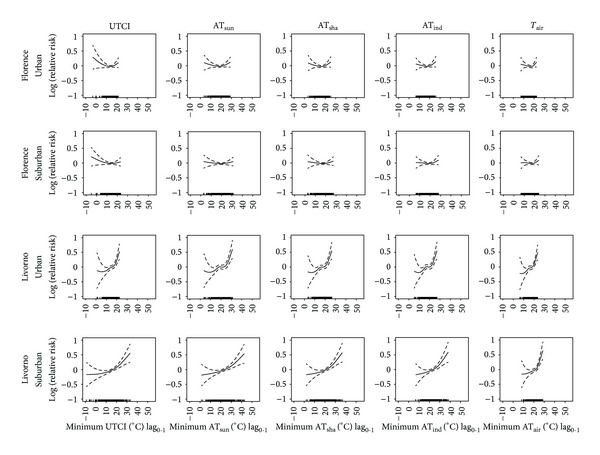
Relationships between daily minimum thermal predictors (lag_0-1_) (°C) and mortality of the very elderly (subjects ≥75 years of age) in an inland city (Florence) and coastal-plain city (Livorno). Relationships estimated by using urban and suburban meteorological data for the period 2006–2008 (May–October). UTCI: Universal Thermal Climate Index; AT_sun_: apparent temperature assessed outdoors also taking the solar radiation contribution into account; AT_sha_: apparent temperature assessed outdoors in the shade; AT_ind_: apparent temperature assessed in indoor conditions; *T*_air_: environmental temperature. Analyses were controlled for air pollution concentrations, daylight hours, year, day of the week, public holidays, and summer population decrement.

The smoothing plots of short-term exposure-response curves during the warmest period of the year (May–October) clearly illustrate the relationships between mortality and the set of independent biometeorological (rational and direct indices) and meteorological (*T*_air_) indicators. The results are reported in Figures 1, 2, and 3 for the average, maximum, and minimum values, respectively. The “U-shaped” relationships between short-term effects (lag_0-1_) of each daily average (Figure 1), maximum (Figure 2), and minimum (Figure 3) thermal indicator and mortality were identified in both urban and suburban areas of the inland plain city. Conversely, prevalent “J-shaped” relationships were identified in the coastal city. In this case, steeper right-hand slope curves (heat effects) were observed which also reached higher relative risks than the inland city.

When the same geographical location was considered, similar exposure-response curves between total mortality and different thermal indicators were usually observed, even if the range of indicators varied depending on specific indicator characteristics. No substantial differences were observed in the curves between urban and suburban areas in the inland city (Figures 1, 2, and 3). On the other hand, different patterns were found between the urban and suburban curves in the coastal city: steeper right-hand slope curves were observed in the urban area (Figures 1, 2, and 3).

The urban daily average AT_sun_ was selected as the best predictive model for the inland plain city. This model showed a high probability (almost 50%) of being the best model among the set of 30 candidate models (Table 4). Furthermore, a significant expected % change in deaths (5.6%, CI: 0.5–10.7, *P* < 0.05) was evidenced. In addition, other models, such as the urban daily average AT_sha_ and maximum UTCI and the urban and suburban minimum AT_sun_, also showed good model-fits, displaying *w*_*i*_(AIC) superior to 5%. However, only the urban daily minimum AT_sun_ still confirmed a significant expected % change of death (6.1%, CI: 1.2–11.0, *P* < 0.05) (Table 4), whereas less model-fits often included the AT_ind_.

The urban daily minimum AT_sha_ was selected as the best predictive model for the coastal city (Table 4). In this case, the probability that this model was the best among all candidate models was almost 60%. A very high significant expected % change in deaths was also observed (10.3%, CI: 4.0–16.6, *P* < 0.01). Furthermore, an elevated probability (21%) of being the second best model among the others was associated with the suburban daily average AT_ind_, even confirming a significant % change in deaths (Table 4). In addition, the urban minimum AT_sun_ and the suburban AT_ind_ also revealed good model-fits, showing *w*_*i*_ (AIC) of 8%. Less predictive model-fits prevalently included daily maximum thermal indicators.

## 4. Discussion

This research provides a substantial contribution to previous studies referred to in identifying the most effective thermal/air temperature predictor of heat-related mortality in different geographical contexts.

A large amount of environmental epidemiological studies carried out worldwide over recent decades applied different thermal indicators by using a predefined relation of changed daily temporal summaries (daily average, maximum, or minimum values), which further complicated the explanations and comparisons of the results.

Most of studies generally used single meteorological parameters, in particular air temperature, or simple, mainly two-parameter, direct indices [3, 6–11] as environmental predictors of mortality. Moreover, recent progress in the field of outdoor thermal comfort assessment led to the development of the UTCI [16], which represents a universal solution to the problem of characterizing the human thermal environment based on the most advanced multinode model of human thermoregulation coupled with a state-of-the-art clothing model. However, at the present time, no consistent information is available regarding the smoothed shape of exposure-response curves between mortality and different biometeorological (direct and rational indices) and air temperature indicators.

Currently, what is clearly known and generally recognized is the short-term impact of heat on mortality with the greatest effect on the elderly [9]. In a recent study carried out in the same geographical context as this study, the authors also evidenced a greater short-term impact of heat on the very elderly population (people aged ≥75) [3]. This is the reason why this study directly focused on the very elderly.

The main findings of this study can be summarized as follows.

- Simple, direct four- and three-parameter indices (AT_sun_ and AT_sha_) showed the highest correlations with UTCI. Conversely, the direct two-parameter index (AT_ind_) and the single meteorological parameter (*T*_air_) showed the lowest correlations.
- “U-” and “J-shaped” relationships between short-term effects of each daily thermal indicator and mortality were identified in the inland and coastal plain cities, respectively. Furthermore, when the same geographical location was considered, similar exposure-response curves were observed between total mortality and different thermal indicators in both urban and suburban areas of the inland plain city. On the other hand, steeper right-hand slope curves (heat effects) were observed in the urban area of the coastal city than in the suburban ones.
- Urban daily average AT_sun_ and minimum AT_sha_ showed the lowest AIC values and the highest probability (almost 50% for the average AT_sun_ and 60% for the minimum AT_sha_) of being the best model among the set of 30 candidate models. For these reasons, these thermal indicators were selected as the best predictors of heat-related all-cause mortality in the very elderly for the inland (urban daily average AT_sun_) and coastal (urban daily minimum AT_sha_) cities, also revealing significant expected % change of death due to a 1°C increase above the 75th percentile. On the other hand, less predictive model-fits often included the AT_ind_ for the inland city and prevalently involved daily maximum indicators for the coastal city.

### 4.1. UTCI versus Direct Indices and Air Temperature Variables

As expected, simple direct indices which also consider the extra effect of wind and solar radiation in addition to relative humidity and air temperature for measuring thermal comfort showed the highest correlations with UTCI. In a recent study [12] the authors also found a significant better fit of three-parameter AT (the version which also accounts for the extra effect of wind speed, called AT_sha_ in this study) with UTCI than other simple two-parameter indices, such as the Heat Index, the Humidex, or the Wet-Bulb Globe Temperature. In particular, the authors observed a correlation coefficient of 95.35%, slightly lower than the mean values of 96.46% and 96.67% highlighted in this study when daily average and minimum values were considered, respectively. However, it was also higher than the correlation coefficient of 88.16% found in this study when daily maximum values were used. Blazejczyk et al. [12] also evidenced a lower slope coefficient, 0.716, than the very good ones detected in this study, 1.000, 1.028, and 0.922, when mean average, minimum, and maximum values were taken into account. This probably depends on the different dataset characteristics of the meteorological variables used in both studies. The correlation and slope coefficients between simple, direct two-parameter indices (Heat Index, Humidex or Wet-Bulb Globe Temperature) and UTCI found in the Blazejczyk et al. [12] study were lower than those observed in this study, probably due to the fact that the thermal indices used required a more restrictive air temperature range of application (calculated for air temperature >20°C) than in this study in which the simple, direct two-parameter index used (AT_ind_) was applicable over a wide range of temperatures (no temperature restrictions were considered).

In conclusion, the authors also reported that direct indices (i.e., AT) are less correlated with UTCI than indices derived from various human heat budget models, such as the Perceived Temperature, the Physiological Equivalent Temperature, or the Standard Effective Temperature. One of the possible reasons for the noncompliance is the lack of the radiation factor in the equations [12]. However, these authors did not apply the four-parameter AT version [24] which also includes the extra effect of solar radiation (AT_sun_). The AT_sun_ revealed the best relationships with UTCI among the thermal indicators considered when daily average and maximum values were used in this study.

The reason behind the higher correlation coefficient of the direct three- and four-parameter indices with UTCI recorded in this study, compared to other more simple, direct two-parameter indices or the individual air temperature, is only due to the inclusion of the extra effect of wind speed in AT_sha_ and the global radiation in AT_sun_. The inclusion of these parameters in the thermal comfort assessment allows a better and more complete representation of the thermal environment as considered when using UTCI. This is also confirmed by the fact that when maximum thermal indices and air temperature indicators were compared with UTCI, the AT_sun_, which also considers the radiant solar contribution for outdoor thermal comfort evaluation, was the only indicator to show a high correlation coefficient value >90%. Conversely, when daily minimum values were considered, the solar radiation contribution assumed less importance and both AT_sun_ and AT_sha_ showed similar high correlations with UTCI. Furthermore, in these conditions, simple two-parameter indices, such as AT_ind_, also showed high correlation coefficients with UTCI (over 90%).

### 4.2. Exposure-Response Curves between Mortality and each Thermal Indicator

Both cities showed two well-known shaped short-term relationships between different thermal indicators and mortality of the very elderly (aged ≥75), already evidenced in many previous studies [3, 34, 35]. In this study, these relationships were studied over a wider “warm” period (from May to October) than that generally considered in previous studies (from June to August) to detect the association between heat and mortality. The reason for this choice was that the area studied is often subjected to “anomalous” heat stress conditions that can also occur earlier, during the average/late spring months (such as May/June) or later, during the early/average autumn months (September/October). By way of example, during May of 2007 and May and September of 2008, the inland plain city also experienced several days with very high (for the period) maximum air temperatures, close to or slightly higher than 27°C. It is known that an equivalent high daily temperature could be more dangerous in May, when people are not still acclimatized to the heat, than in August, because very high temperatures are unusual that early in the season [36].

Exposure-response curves between total mortality and different thermal indicators clearly showed different shapes at a regional scale, with stepper right-hand slope curves (heat effects on mortality) in the milder coastal plain city. These patterns were already evidenced in a previous study [3] and the authors concluded that a population living at the coast is more susceptible to the heat and less adaptable to sudden temperature changes and heat extremes. This is because the coastal city generally shows reduced daily temperature ranges and lower temperature variations during the warmest period of the year than the inland city.

As already reported in a previous Korean study [15], similar exposure response curves between all-cause mortality and a simple daily mean, maximum, and minimum direct index (the AT_ind_) and *T*_air_ were observed in two different Korean cities (one inland and one seashore cities). This is in agreement with our study, where the curves obtained with different indicators had similar shapes. However, the Korean study also showed different curves when another rational index (the Perceived Temperature) was used as an indicator. This situation has not been confirmed in this study, where a more advanced rational index (UTCI) was applied.

Our study also showed that all thermal rational and direct indices assessed and the air temperature measured by using meteorological data recorded by weather stations located in urban and suburban areas might have heterogeneous relationships with mortality in the coastal city. In another Italian study [37] which evaluated the association between mortality and heat measured by using airport and city-centre temperatures, the authors found that in two cities (Rome and Turin) the exposure values were very similar, while in another city (Milan) the AT differed greatly between stations. Therefore, it is also plausible to expect dissimilar heat-related mortality results when different sources of meteorological data are used in epidemiological environmental studies. For this reason, studies assessing the potential impact of heat on mortality need to take different environmental exposure contexts into account, that is, meteorological data recorded by urban or suburban weather stations, in order to ensure more accurate estimates of health effects on the population.

### 4.3. The Selection of the Best Thermal Indicator of Heat-Related Mortality in Different Geographical Areas

At the present time, only a few recent studies have tried to investigate the effect of various thermal indictors on mortality data [14, 15, 17–19] in the aim of selecting the best predictor of mortality. Currently, great uncertainty surrounds the establishing of the most appropriate thermal indicator, as well as the daily temporal characteristic (daily average, maximum, or minimum values) for best fitting the thermal impact on mortality.

A previous study carried out in US cities [17] and two in Australian cities [18, 19] found that no single temperature measure was superior to the others. One reason advanced by several authors [17] was the high correlation among different thermal indicators showing the same predictive ability. However, these studies only used individual meteorological parameters or simple, direct two- or three-parameter indices as potential indicators of mortality. Furthermore, the analyses were not controlled for air pollution in the American study, while the Australian studies controlled for PM_10_, NO_2_, and O_3_. In another study carried out in two Korean cities [15], the authors investigated the effect on mortality, also controlled for PM_10_ and O_3_, by using a rational index, the Perceived Temperature based on a human heat budget model, compared with other simple direct indices or air temperature indicators. The authors found that in terms of model fitting by AIC, for one city (Seoul), the daily maximum Perceived Temperature was the best predictor for the all-cause mortality risk. The interpretation of the authors is that, because the city of Seoul is located near the sea, the Perceived Temperature, which also includes the humidity effect, was a better indicator of excess mortality than the air temperature. This is also confirmed by the fact that the two other best model indicators found for Seoul always accounted for the humidity effect (daily mean Perceived Temperature and minimum AT). Conversely, the worst indicator of total mortality included the daily maximum air temperature. However, because different results were also observed for another city, the authors concluded that the rational and direct indices used in the study do not always act as the best predictors for the assessment of heat-related mortality [15]. In a more recent study [14] conducted in Taiwan, the authors identified the apparent temperature index, assessed in the form of a three-parameter AT index, as the most optimal high-temperature index associated with all-cause mortality. The AT was selected as the best model from among eight high-temperature indices, including three single air temperature measurements (average, maximum, and minimum) and five different simple, direct indices.

In this study, our findings partially confirm several results of the Korean study and are in firm agreement with the Lin et al. [14] conclusion. Indeed, direct multivariate indices (AT_sun_ and AT_sha_), which also account for the extra effect of wind speed and/or solar radiation, as well as the combined contribution of humidity and air temperature, were selected as the best predictors for all-cause very-elderly mortality risk in the inland plain city (daily average AT_sun_) and the coastal city (daily minimum AT_sha_). In particular, the probability of being the best predictive model among the other candidate models considered in this study was nearly (in the inland plain city) or above (in the coastal plain city) 50%.

However, the UTCI was never identified as the best thermal predictor of all-cause very-elderly mortality in this study. This is probably due to the specific characteristics of UTCI, which includes a complex multinode model of thermoregulation coupled with a clothing model that determines strong thermal sensitivity greatly influenced by the immediate surroundings. For this reason, the UTCI naturally represents the best approach for exhaustive thermal comfort studies. However, it also requires accurate micrometeorological measurements of a specific location and subjective information (clothing thermal and physical activity characteristics) which is not generalized for epidemiological studies over wide geographical areas (such as cities), where great, uncontrolled variability and different types of human behavior exist. For this reason simplified approaches for thermal comfort assessment for a wide set of people have been preferred.

However, although the use of direct multivariate (four- or three-parameter) indices revealed the best fitting with all-cause very-elderly mortality attributable to heat stress, different multivariate index indicators were identified in both cities. For example, the urban average AT_sun_, identified as the best predictive model for the inland plain city, also showed a poor predictive power for the coastal city. The same was also observed for the best indicator identified for the coastal city. Consequently, our findings suggest the use of direct multivariate indices as indicators naturally recommended for epidemiological heat-related mortality studies especially when the very elderly are considered; however, the choice of the most appropriate multivariate thermal index should be based on the geographical characteristics of the place investigated and the data available. In the inland plain city, the use of a full direct index is suggested which also accounts for the extra solar radiation contribution, besides the other meteorological effects. In other terms, because the extra effect of wind speed and relative humidity is more prevalent in a coastal city than in an inland city, the use of three- (*T*_air_, RH, and *V*_10_) or also direct two-parameter (*T*_air_ and RH) indices is sufficient for obtaining an effective prediction of heat-related mortality.

Furthermore, this study also showed that thermal indices measured by an urban meteorological station in both cities had a greater relationship with heat-related mortality than thermal indices measured by a suburban station, even though no real break exists between urban and suburban areas. However, it was not possible to identify an unequivocal daily temporal characteristic (daily average, maximum, or minimum values) of thermal index measurements for both cities. Indeed, daily average and minimum thermal index indicators were identified as the best models in the inland and coastal cities, respectively. Several researchers [38] reported that the use of the daily average temperature as an exposure indicator of thermal conditions, taking the whole day and night into account, generally provides more easily interpreted results within a policy context. In another study [39], the authors also found a close association of the minimum temperature with heat effects. The impact might also be the strongest when the very elderly are considered: when heat discomfort conditions also persist during night-time hours, when the body generally requires physiological rest, and when renal tubular conservation of sodium and water diminishes during periods of dehydration, all of which represent aggravating factors for health, especially in the “elderly frail” with a significant increase in the mortality risk. Conversely, daily maximum indicators represent the worst model-fits in the coastal city. This is probably due to the fact that cities on the coast generally have reduced daily temperature ranges and less frequent daily “extremes” than inland cities.

### 4.4. Strengths and Limitations

This study has several strengths. Firstly, the newly developed UTCI, which represents the state-of-the-art of outdoor thermal comfort assessment, has been included, together with other thermal indices, as a potential predictor of heat-related mortality. Secondly, an accurate model selection was carried out by using the Akaike weight scheme which is a useful tool for supplementing the basic results obtained with the AIC model comparison analysis [31]. The analyses were controlled for the main air pollution concentrations as confounding effects. Previous studies reported differences between models with and without the adjustment of air pollution, especially in the case of extremely hot days and vulnerable groups [40, 41].

Some limitations should also be pointed out. Since the identification of the best thermal predictor of heat-related mortality was only investigated in two cities in this study, the results cannot be applied to other countries and/or climates. Indeed, the interaction between weather and human health is mediated by socioeconomical and cultural (i.e., clothing and diet) factors, besides adaptation of the population to the local climate. However, it should also be noted that the two cities considered in this study had two clearly different climate conditions. In a recent study [42] the authors showed how elderly patients who are living alone and also using community care services are two times more at heat-related health risk than other elderly people. Living alone, also associated with critical health and low socioeconomic status, can generally result in a significant increase in vulnerability to heat conditions. Further improvements could be provided in heat-related health risk estimations by taking socio-economic conditions, perceptions, and cost factors of using air-conditioning at home during the warmest period of the year and cultural differences into consideration.

In addition, the analyses could also be extended to different mortality categories and age groups. In this way it would be possible to understand if different age and mortality categories are sensitive to different thermal indicators.

## 5. Conclusion

This study showed how the use of direct multivariate indices, which also account for the extra effect of wind speed and/or solar radiation as well as the combined contribution of air humidity and temperature, revealed the best fitting with all-cause very-elderly mortality attributable to heat stress. However, different multivariate thermal index predictors were identified in both inland and coastal plain cities. In addition, UTCI, which represents the best performing index in thermal comfort assessment, was never identified as the best predictor of all-cause very-elderly mortality. It is hypothesized that simplified approaches for a general thermal comfort assessment, such as direct multivariate thermal indices, are preferable for epidemiological purposes. The choice of the most appropriate multivariate thermal index as a predictor of heat-related mortality should be based on geographical characteristics and the availability data regarding the area considered. Further studies are needed to confirm these results. The better understanding of the impact of different thermal indicators on mortality in different geographical contexts will provide relevant information for developing efficient public health programs and heat-related health risk assessments. This information could prove to be very useful in developing preventive measures and for functional implementation to improve previous local public health emergency plans related to heat conditions.

## Figures and Tables

**Table 1:** Descriptive statistics for daily all-cause mortality of the very elderly (subjects ≥75 years of age), air pollution, and meteorological parameters measured in urban and suburban areas of Florence and Livorno during the warmest period of the year (May–October) from 2006 to 2008. Mean, standard deviation (SD), and 10th, 75th, and 90th percentiles are shown.

Variables	Unit	Inland plain city: Florence				Coastal plain city: Livorno			
		Mean (±SD)	10th	75th	90th	Mean (±SD)	10th	75th	90th
Mortality age ≥75 years	N	7.0 (±2.7)	4	9	11	3.5 (±2.0)	1	5	6
Air pollution									
SO_2_	*µ*g m^−3^	1.6 (±0.8)	0.8	1.9	2.6	4.3 (±3.9)	0.7	6.0	9.5
NO_2_	*µ*g m^−3^	38.1 (±11.9)	23.5	45.4	52.8	31.5 (±7.2)	23.4	35.2	40.1
CO	*µ*g m^−3^	0.6 (±0.2)	0.4	0.7	0.8	0.5 (±0.1)	0.4	0.6	0.7
O_3_	*µ*g m^−3^	70.1 (±20.4)	42.9	83.3	95.7	76.0 (±18.0)	52.1	87.9	99.4
PM_10_	*µ*g m^−3^	30.6 (±11.3)	18.6	35.3	43.4	27.7 (±7.9)	19.4	31.2	37.4
*Meteorology *									
Urban									
*T*_air_	°C	20.6 (±3.8)	15.6	23.9	25.7	20.4 (±3.3)	16.1	23.2	24.7
RH	%	40.1 (±13.3)	24.6	48.2	57.2	50.0 (±12.6)	31.9	58.4	64.5
*V*_10_	m s^−1^	1.7 (±0.7)	0.9	1.9	2.2	2.0 (±0.6)	1.1	2.3	2.9
*T*_mrt_	°C	21.1 (±5.9)	12.7	25.7	28.1	21.2 (±5.1)	13.8	25.1	26.9
Suburban									
*T*_air_	°C	21.9 (±4.2)	16.4	25.5	27.8	22.6 (±3.6)	17.9	25.4	27.3
RH	%	42.4 (±13.2)	25.8	50.0	59.6	61.1 (±16.6)	39.4	72.4	84.1
*V*_10_	m s^−1^	3.1 (±0.7)	2.2	3.5	3.9	5.5 (±2.5)	3.5	5.8	8.3
*T*_mrt_	°C	22.5 (±6.3)	13.6	27.3	30.0	23.2 (±5.2)	16.0	26.9	29.1

*T*_air_: environmental temperature; RH: relative humidity; *V*_10_: 10 m high horizontal wind speed; *T*_mrt_: mean radiant temperature.

**Table 2:** Descriptive statistics of daily thermal indices and air temperature indicators assessed and measured in urban and suburban areas of Florence and Livorno during the warmest period of the year (May–October) from 2006 to 2008. Mean, standard deviation (SD), and 10th, 75th, and 90th percentiles are shown.

Daily thermal indices and air temperature indicators (°C)	Inland plain city: Florence				Coastal plain city: Livorno			
	Mean (±SD)	10th	75th	90th	Mean (±SD)	10th	75th	90th
*Urban *								
Average								
UTCI	20.6 (±4.3)	15.0	24.0	26.0	20.8 (±3.9)	15.7	24.0	25.5
AT_sun_	24.6 (±5.3)	17.6	28.7	31.5	25.2 (±5.1)	18.6	29.2	31.6
AT_sha_	20.7 (±4.5)	14.8	24.3	26.6	21.1 (±4.2)	15.6	24.6	26.4
AT_ind_	21.3 (±4.2)	15.8	24.4	26.7	21.7 (±4.0)	16.5	25.0	26.7
*T*_air_	20.6 (±3.8)	15.6	23.9	25.7	20.4 (±3.3)	16.1	23.3	24.7
Minimum								
UTCI	13.7 (±3.7)	9.0	16.5	18.1	14.6 (±3.6)	10.1	17.3	18.8
AT_sun_	18.2 (±4.8)	11.7	21.8	23.9	19.3 (±4.8)	13.1	23.0	25.1
AT_sha_	16.0 (±4.1)	10.5	19.1	21.1	17.1 (±4.1)	11.8	20.2	22.0
AT_ind_	16.8 (±3.9)	11.5	19.8	21.6	17.9 (±3.9)	12.9	20.8	22.5
*T*_air_	15.8 (±3.3)	11.3	18.3	20.0	16.7 (±3.2)	12.5	19.1	20.7
Maximum								
UTCI	30.9 (±5.1)	23.7	34.9	37.4	30.8 (±4.2)	25.3	33.9	35.9
AT_sun_	33.1 (±6.2)	24.7	38.0	41.0	34.4 (±5.9)	26.8	39.2	41.6
AT_sha_	25.7 (±5.2)	18.9	29.9	32.7	25.6 (±4.5)	20.0	29.1	31.6
AT_ind_	25.9 (±4.7)	19.7	29.7	32.1	25.9 (±4.3)	20.7	29.4	31.5
*T*_air_	26.0 (±4.7)	20.1	29.9	32.4	24.9 (±3.7)	20.7	28.1	29.9

*Suburban *								
Average								
UTCI	21.2 (±5.1)	14.6	25.3	28.0	21.1 (±5.8)	13.8	25.2	28.0
AT_sun_	25.9 (±6.0)	18.1	30.5	33.7	27.7 (±6.8)	19.2	32.5	36.0
AT_sha_	22.0 (±5.1)	15.4	26.0	28.9	23.4 (±5.6)	16.4	27.6	30.3
AT_ind_	23.0 (±4.7)	16.9	26.7	29.3	25.6 (±5.2)	18.9	29.3	32.1
*T*_air_	21.9 (±4.2)	16.4	25.5	27.8	22.6 (±3.6)	17.9	25.4	27.3
Minimum								
UTCI	13.8 (±4.5)	8.2	17.2	19.4	14.8 (±6.6)	6.6	19.6	22.6
AT_sun_	19.2 (±5.3)	12.1	23.2	26.0	22.3 (±6.8)	13.8	27.0	30.1
AT_sha_	16.8 (±4.7)	10.7	20.3	22.8	19.6 (±5.8)	12.3	23.7	26.8
AT_ind_	18.1 (±4.4)	12.4	21.3	23.9	22.4 (±5.2)	15.6	25.9	29.0
*T*_air_	16.6 (±3.7)	11.7	19.3	21.4	19.8 (±3.8)	14.7	22.8	24.8
Maximum								
UTCI	30.7 (±6.0)	22.4	35.3	37.9	28.5 (±5.4)	21.6	32.4	34.7
AT_sun_	33.5 (±6.8)	24.1	38.7	41.9	34.3 (±7.2)	24.8	39.8	43.2
AT_sha_	26.8 (±5.7)	19.3	31.5	34.3	27.0 (±5.4)	20.0	30.9	33.7
AT_ind_	27.6 (±5.3)	20.7	31.9	34.6	28.4 (±5.2)	21.6	32.4	34.9
*T*_air_	27.4 (±5.1)	20.6	31.5	34.0	25.1 (±3.5)	20.6	27.7	29.9

UTCI: Universal Thermal Climate Index; AT_sun_: apparent temperature assessed outdoors also taking into consideration the solar radiation contribution; AT_sha_: apparent temperature assessed outdoors in the shade; AT_ind_: apparent temperature assessed in indoor conditions; *T*_air_: environmental temperature.

**Table 3:** Correlation analyses between daily average, minimum, and maximum UTCI versus direct thermal indices and air temperature indicators in urban and suburban areas of Florence and Livorno during the warmest period of the year (May–October) from 2006 to 2008.

Daily thermal indices and air temperature indicators	Inland plain city: Florence		Coastal plain city: Livorno		Mean values from all stations
	Urban (missing data = 0%)	Suburban (missing data = 0.4%)	Urban (missing data = 0%)	Suburban (missing data = 0%)	Slope; *R* ^2^ (%)
	Slope; *R* ^2^ (%)	Slope; *R* ^2^ (%)	Slope; *R* ^2^ (%)	Slope; *R* ^2^ (%)	
Average					
AT_sun_	1.193; 96.22	1.121; 96.85	1.284; 96.41	1.114; 96.51	1.178; 96.50
AT_sha_	1.029; 96.27	0.971; 96.76	1.073; 96.10	0.926; 96.71	1.000; 96.46
AT_ind_	0.949; 95.00	0.891; 94.61	1.008; 94.80	0.821; 88.95	0.917; 93.34
*T*_air_	0.865; 96.07	0.796; 94.82	0.827; 94.62	0.562; 87.24	0.763; 93.19
Minimum					
AT_sun_	1.280; 97.32	1.160; 95.26	1.293; 97.43	0.992; 96.21	1.192; 96.56
AT_sha_	1.113; 97.41	1.014; 94.82	1.138; 97.99	0.848; 96.46	1.028; 96.67
AT_ind_	1.038; 96.07	0.935; 90.50	1.070; 96.11	0.711; 83.53	0.939; 91.55
*T*_air_	0.866; 93.50	0.764; 87.95	0.854; 92.60	0.502; 79.27	0.747; 88.33
Maximum					
AT_sun_	1.110; 93.61	1.050; 94.03	1.263; 91.80	1.176; 89.51	1.150; 92.24
AT_sha_	0.951; 91.06	0.909; 92.49	0.960; 85.96	0.869; 83.11	0.922; 88.16
AT_ind_	0.863; 89.77	0.830; 90.59	0.891; 84.15	0.792; 76.15	0.844; 85.17
*T*_air_	0.881; 93.58	0.824; 93.59	0.801; 87.70	0.549; 76.63	0.764; 87.88

UTCI: Universal Thermal Climate Index; AT_sun_: apparent temperature assessed outdoors also taking into consideration the solar radiation contribution; AT_sha_: apparent temperature assessed outdoors in the shade; AT_ind_: apparent temperature assessed in indoor conditions; *T*_air_: environmental temperature.

**Table 4:** Summary of predictive model fits and expected % change in deaths due to a 1°C increase in the thermal predictor over the 75th percentile based on each thermal indicator. *P < 0.05; **P < 0.01.

Inland plain city: Florence				Coastal plain city: Livorno			
Indicators	AIC	*w*_*i*_ (AIC)	% change	Indicators	AIC	*w*_*i*_ (AIC)	% change
Ave_urb_AT_sun_	663.3	0.48	5.6 (0.5; 10.7)*	Min_urb_AT_sha_	587.7	0.58	10.3 (4.0; 16.6)**
Ave_urb_AT_sha_	665.1	0.19	4.8 (−1.1; 10.7)	Ave_sub_AT_ind_	589.7	0.21	4.7 (0.2; 9.2)*
Max_urb_UTCI	666.2	0.11	4.9 (−1.0; 10.8)	Min_urb_AT_sun_	591.7	0.08	6.8 (1.7; 11.9)**
Min_urb_AT_sun_	667.2	0.07	6.1 (1.2; 11.0)*	Min_sub_AT_ind_	591.7	0.08	4.8 (0.7; 8.9)*
Min_sub_AT_sun_	667.9	0.05	3.8 (−0.3; 7.9)	Max_sub_AT_ind_	594.7	0.02	4.5 (−0.4; 9.4)
Min_urb_UTCI	668.2	0.04	9.2 (2.1; 16.3)*	Ave_sub_AT_sun_	596.0	0.01	3.3 (−0.4; 7)
Ave_urb_*T*_air_	669.2	0.03	3.8 (−3.8; 11.4)	Ave_sub_AT_sha_	596.5	0.01	4.7 (0.1; 9.4)*
Max_urb_AT_ind_	671.0	0.01	2.7 (−2.8; 8.2)	Min_sub_AT_sun_	597.5	0.00	4.1 (0.8; 7.4)*
Max_sub_UTCI	671.5	0.01	4.3 (−0.6; 9.2)	Max_sub_UTCI	599.0	0.00	6.5 (1.4; 11.6)*
Min_urb_AT_ind_	672.7	0.00	8.0 (1.9; 14.1)*	Min_urb_AT_ind_	600.2	0.00	9.6 (3.3; 15.9)**
Max_urb_*T*_air_	673.2	0.00	1.3 (−4.8; 7.4)	Min_sub_UTCI	602.4	0.00	4.9 (0.8; 9.0)*
Max_sub_*T*_air_	673.5	0.00	2.3 (−3.2; 7.8)	Max_sub_AT_sun_	603.4	0.00	2.6 (−1.1; 6.3)
Ave_sub_*T*_air_	674.0	0.00	1.5 (−5.4; 8.4)	Ave_urb_AT_ind_	604.4	0.00	8.5 (1.4; 15.6)*
Max_urb_AT_sha_	674.8	0.00	3.2 (−2.1; 8.5)	Min_sub_AT_sha_	604.9	0.00	4.7 (0.6; 8.8)*
Ave_sub_AT_ind_	675.7	0.00	4.0 (−1.1; 9.1)	Max_sub_AT_sha_	605.6	0.00	3.6 (−1.3; 8.5)
Max_sub_AT_sha_	676.1	0.00	4.4 (−0.5; 9.3)	Min_urb_UTCI	606.7	0.00	11.7 (4.1; 19.3)**
Max_urb_AT_sun_	676.4	0.00	2.8 (−1.9; 7.5)	Ave_sub_*T*_air_	607.7	0.00	8.4 (0.1; 16.8)*
Min_sub_UTCI	677.9	0.00	3.2 (−1.9; 8.3)	Max_urb_AT_ind_	609.1	0.00	11.7 (3.9; 19.5)**
Min_sub_AT_sha_	678.4	0.00	5.3 (0.4; 10.2)*	Ave_sub_UTCI	613.8	0.00	5.5 (1.0; 10.0)*
Ave_sub_UTCI	679.3	0.00	3.3 (−2.2; 8.8)	Ave_urb_UTCI	617.6	0.00	11.3 (2.7; 19.9)**
Min_urb_*T*_air_	679.8	0.00	10.2 (1.5; 16.7)*	Min_urb_*T*_air_	617.8	0.00	9.2 (0.4; 18.0)*
Ave_sub_AT_sha_	680.0	0.00	3.0 (−1.9; 7.9)	Ave_urb_AT_sha_	620.6	0.00	6.4 (−0.5; 13.3)
Ave_sub_AT_sun_	680.4	0.00	3.7 (−0.4; 7.8)	Min_sub_*T*_air_	621.3	0.00	7.2 (−0.4; 14.8)
Min_urb_AT_sha_	680.9	0.00	7.5 (1.6; 13.4)*	Ave_urb_AT_sun_	621.8	0.00	6.5 (1.0; 12.0)*
Max_sub_AT_sun_	681.8	0.00	4.3 (0.2; 8.4)*	Max_urb_AT_sun_	622.7	0.00	6.2 (0.7; 11.7)*
Max_sub_AT_ind_	685.0	0.00	4.4 (−0.5; 9.3)	Max_sub_*T*_air_	623.2	0.00	2.8 (−4.3; 9.9)
Ave_urb_UTCI	691.8	0.00	5.6 (−1.1; 12.3)	Ave_urb_*T*_air_	631.4	0.00	10.2 (0.8; 19.6)*
Min_sub_*T*_air_	702.3	0.00	7.1 (0.6; 13.6)*	Max_urb_UTCI	639.2	0.00	9.3 (1.1; 17.5)*
Min_sub_AT_ind_	704.8	0.00	5.3 (0.2; 10.4)*	Max_urb_*T*_air_	641.5	0.00	4.8 (−3.6; 13.2)
Ave_urb_AT_ind_	713.8	0.00	4.5 (−1.0; 10.0)	Max_urb_AT_sha_	642.1	0.00	7.2 (0.3; 14.1)*

AIC: Akaike's Information Criterion; *w*_*i*_ (AIC): Akaike weights; Max: maximum; Min: minimum; Ave: average; urb: urban; sub: suburban; UTCI: Universal Thermal Climate Index; AT_sun_: apparent temperature assessed outdoors also taking into consideration the solar radiation contribution; AT_sha_: apparent temperature assessed outdoors in the shade; AT_ind_: apparent temperature assessed in indoor conditions; *T*_air_: environmental temperature.

## Abbreviations

UTCI: Universal Thermal Climate Index
AIC: Akaike's Information Criterion
*w*_*i*_ (AIC): Akaike weights
AT: Apparent temperature
AT_sun_: Apparent temperature assessed outdoors, also taking the solar radiation contribution into consideration
AT_sha_: Apparent temperature assessed outdoors in the shade
AT_ind_: Apparent temperature assessed in indoor conditions
*T*_air_: Environmental temperature
RH: Relative humidity
*e*: Water vapor pressure
*V*_10_: 10 m high horizontal wind speed
GR: Global radiation.
